# Relationship between TLR4 and NF-κB p65 protein expressions and clinical radiosensitivity of patients with esophageal squamous cell carcinoma

**DOI:** 10.12669/pjms.305.5472

**Published:** 2014

**Authors:** Hua Tang, Feng Wang, Xi-Fa Zhou, Jian Zhou, Ling Chen, Ju-Dong Luo, En-Ci Xu

**Affiliations:** 1 Hua Tang, Department of Radiotherapy Oncology, Changzhou Fourth People’s Hospital, 68 Honghe Road, Changzhou 213032, Jiangsu Province, P. R. China.; 2 Feng Wang, Department of Radiotherapy Oncology, Changzhou Fourth People’s Hospital, 68 Honghe Road, Changzhou 213032, Jiangsu Province, P. R. China.; 3 Xi-Fa Zhou, Department of Radiotherapy Oncology, Changzhou Fourth People’s Hospital, 68 Honghe Road, Changzhou 213032, Jiangsu Province, P. R. China.; 4 Jian Zhou, Department of Radiotherapy Oncology, Changzhou Fourth People’s Hospital, 68 Honghe Road, Changzhou 213032, Jiangsu Province, P. R. China.; 5 Ling Chen, Department of Radiotherapy Oncology, Changzhou Fourth People’s Hospital, 68 Honghe Road, Changzhou 213032, Jiangsu Province, P. R. China.; 6 Ju-Dong Luo, Department of Radiotherapy Oncology, Changzhou Fourth People’s Hospital, 68 Honghe Road, Changzhou 213032, Jiangsu Province, P. R. China.; 7 En-CiXu, Department of Radiotherapy Oncology, Changzhou Fourth People’s Hospital, 68 Honghe Road, Changzhou 213032, Jiangsu Province, P. R. China.

**Keywords:** Esophageal cancer, Radiotherapy, TLR4, NF-κB

## Abstract

***Objective:*** To study the relationship between TLR4 and NF-κB p65 protein expressions in tumor tissues after radiotherapy and clinical radiosensitivity of patients with esophageal squamous cell carcinoma.

***Methods:*** A total of 93 patients with esophageal squamous cell carcinoma first treated in our hospital by radiotherapy and surgeries from November 2010 to December 2013 were selected. They were then divided into a severe reaction group, a moderate reaction group and a mild reaction group according to the postoperative pathological examination results of tumor tissues. The expressions of TLR4 and NF-κB p65 in the tumor samples were detected by Western blotting.

***Results:*** Compared with the severe reaction group, the expression levels of TLR4 and NF-κB p65 in the moderate reaction group significantly increased (P<0.05). Similarly, the expression levels of the mild reaction group were significantly higher than those of the moderate reaction group (P<0.05).

***Conclusion: ***Reducing the expression levels of TLR4 and NF-κB p65 proteins may increase the radiosensitivity of patients with esophageal cancer.

## INTRODUCTION

China has the highest morbidity and mortality rates of esophageal cancer worldwide, of which squamous-cell carcinoma is most common. Most patients have entered the middle or advanced stage upon diagnosis, thus making the prognosis very unsatisfactory.^1^ Currently, esophageal cancer is mainly treated by combined radiotherapy, chemotherapy, biotherapy and surgery, among which radiotherapy is a well-established postoperative method to effectively simplify surgical procedure and to improve the resection rate. Besides, multi-center retrospective and prospective studies have verified that postoperative radiotherapy could evidently decrease regional recurrence rate and odds of lymph node metastasis.^2^ However, some patients are prone to resisting radiotherapy. Therefore, it is of great clinical significance to uncover the molecular mechanism of resistance of tumor tissues to radiotherapy and to propose individualized treatment plans for esophageal cancer. In this study, the relationship between TLR4 and NF-κB p65 protein expressions in tumor tissues after radiotherapy and clinical radiosensitivity of patients with esophageal squamous cell carcinoma was explored. The results provide valuable evidence for clinical treatment of esophageal cancer.

## METHODS

***General information: ***A total of 93 patients with squamous cell carcinoma in the middle thoracic esophagus (regional advanced stage, T3-4N0-1), who were first treated in our hospital by radiotherapy and surgeries from November 2010 to December 2013, were selected. They all received radiotherapy before surgery. This study was approved by the institutional ethics committee of our hospital, and written consent was obtained from all patients. Exclusion criteria: The patients who had received chemotherapy and immunotherapy were excluded.

***Postoperative radiotherapy methods: ***The patients received radiotherapy at primary tumors and mediastinal lymphatic drainage areas by using SIEMENS/PRIMUS accelerator (6MXX) 6 MV-X rays. Double-spiral CT was used for targeting, and image transmission CMS XIO 4.6.4 system was employed to prepare treatment plans. Gross tumor volume (GTV) and clinical target volume (CTV) were defined by ICRU Report 50. GTV included esophageal tumors and surrounding swollen lymph nodes, and CTV included suspicious infiltrates and mediastinal lymphatic drainage areas around the esophageal tumor. In addition, a 5 mm planning target volume (PTV) outwards was generated. According to intensity-modulated radiation therapy protocol, the patients were treated with the dose of DT to 40 Gy/20 f/26 d, 2-3 weeks after which thoracotomy was performed.

***Diagnostic standards for radiosensitivity: ***Paraffin-embedded tumor tissues were subjected to HE staining according to standard operating procedure. The postoperative reactions were classified according to pathological standards and previous literature.^3^ (1) Mild reaction: Mild degenerative changes of tumor cells, decreased nuclear division, and infiltration of a small number of inflammatory cells and angiogenesis in tumors; (2) moderate reaction: disappearance of most tumor cells, and degenerative changes of residual tumors that were wrapped by granulation tissues; (3) severe reaction: complete disappearance of tumor cells, fibrosis in the tumor bed, reduced blood vessels, chronic infiltration of inflammatory cells, and scarring.

***Western blotting: ***The tumors resected during surgery were transferred in liquid nitrogen immediately, and stored in a -80°C refrigerator. Tumor tissues (100 mg) were collected from each case, the nuclear and total proteins in which were extracted according to the instructions of corresponding kits. The proteins were quantified by the BCA method. Protein samples (35 μg) were transferred onto a film after electrophoresis and blocked by 5% skimmed milk powders for 1 h. β-Actin, H3, NF-κB p65 and TLR4 antibodies were diluted by 5% milk (1:200), with which the film was incubated at 4°C overnight, washed with TBST and rabbit-anti goat secondary antibody diluted by 5% milk. Afterwards, the film was subjected to Western blotting according to ECL instructions by using 1:1 luminescent working solution, and gray scale values were analyzed by Image J software.

***Statistical analysis: ***All data were analyzed by SPSS 13.0. The numerical data were subjected to χ^2^ test, and the categorical data were subjected to univariate analysis of variance. P<0.05 was considered statistically significant.

## RESULTS

***Relationship between radiosensitivity and clinical pathological characteristics: ***The age, gender and tumor length of the three groups were similar (P>0.05), but their differentiation degree, clinical staging and lymphatic metastasis were statistically significantly different (P<0.05). The patients with early clinical staging, high differentiation degree and without lymphatic metastasis had better outcomes (Table-I).

**Fig.1:**
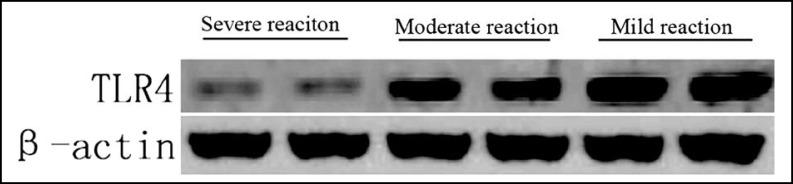
Western blotting of TLR4 protein

**Fig.2:**
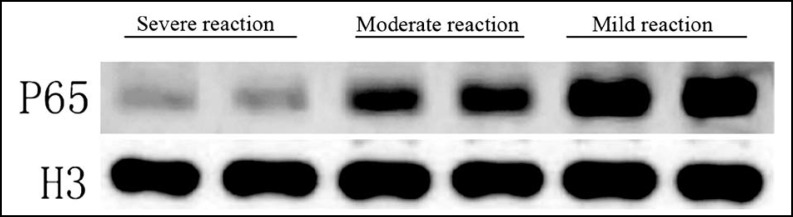
Western blotting of NF-κB p65 protein

***TLR4 and NF*** *-* ***κB p65 protein expressions: ***Compared with the severe reaction group, TLR4 and NF-κB p65 protein expressions in the moderate and mild reaction groups significantly increased (P<0.05) (Fig.1, Fig.2, Table-II).

## DISCUSSION

It has been demonstrated that postoperative radiotherapy can degenerate esophageal cancer cells, decrease the proliferative activity and reduce the tumor volume, thus leading to vascular thrombosis and eliminating micrometastases. As a result, the odds of regional recurrence can be lowered, and subclinical lesions around the tumor can be controlled. Moreover, some patients may enjoy radical resections. Regional recurrence of esophageal cancer usually occurs at the periphery of the original tumor, which can mainly be ascribed to the unradical resection of tumor cells that have invaded adjacent organs and tissues. Such cells may, although highly sensitive to radiation due to high proliferative activity and oxygenation status,^4^ endanger the patients who are not sensitive to radiotherapy. However, the mechanism for radiation resistance remains unclear. Hence, it is of great clinical significance to improve the prognosis by minimizing radiation resistance and by preparing individualized treatment plans. In this study, radiosensitivity was associated with differentiation degree of tumor tissue, clinical staging and lymphatic metastasis. After verifying the individual differences of radiosensitivity, the expressions of TLR4 and p65 were subjected to semiquantitative analyses to elucidate the molecular biological mechanism.

**Table-I:** General information and pathological characteristics

		***Case number***	***Severe reaction group***	***Moderate reaction group***	***Mild reaction group***	***P value***
Case No.		93	30	38	25	
Gender	Male	65	21	25	19	0.688
	Female	28	9	13	6	
Age (years old)	<45	20	7	8	5	0.952
	≥45	73	23	30	20	
Differentiation degree	Low	25	4	8	13	0.016
	Medium	44	16	21	7	
	High	24	10	9	5	
Clinical staging	I,IIa, IIb	40	19	17	4	0.002
	III,IV	53	11	21	21	
Lymphatic metastasis	Yes	53	10	25	18	0.006
	No	40	20	13	7	
Tumor length	<5 cm	50	13	20	17	0.185
	≥5 cm	43	17	18	8	

**Table-II:** Western blotting results

	***Severe reaction group***	***Moderate reaction group***	***Mild reaction group***
TLR4 (/β-actin)	0.07±0.01	0.59±0.11*	0.89±0.16*#
P65 (/H3)	0.05±0.08	0.31±0.08*	0.98±0.23*#

* P<0.05, compared with the severe reaction group;

# P<0.05, compared with the moderate reaction group.

It has previously been confirmed that TLR4 was closely related with the lymphatic metastasis and clinical staging of esophageal cancer patients.^5^ In general, the patients with high TLR4 expressions survived significantly shorter than those with low expressions and are significantly more prone to recurrence.^6^ Once TLR4 receptor is activated by ligand, it exerts biological effects by activating nuclear transcription factor NF-κB that can be activated by several DNA damage pathways, inhibit cell apoptosis, and dominate in the tumor radiation injury pathway.^7^ NF-κB mainly comprises Rel (cRel) and p65 (RelA) subunits, and p65 mainly exerts functional effects. IκB, the inhibitor of NF-κB, binds NF-κB through specific ankyrin repeats of the C-terminus. Meanwhile, IκB suppresses NF-κB from transferring to the nucleus by covering the nuclear localization region. Under resting conditions, NF-κB and IκB coexist as an inactive compound in the cytoplasm. Upon extracellular stimulation, however, IκB is phosphorylated owing to the activation of IκB kinase, which thus exposes the nuclear localization site of NF-κB. Accordingly, free NF-κB rapidly migrates to the cell nucleus and binds specific κB sequences to induce the transcription of relevant genes.^8^ NF-κB can bind DNA more actively in esophageal tumor tissues. In the meantime, significantly more RelA is expressed in tumor tissues than that in paracancerous tissues, while significantly less IκB is expressed. Besides, increase in the activity of NF-κB can induce the drug tolerance of esophageal tumor cells,^9^ so inhibiting its activity is able to enhance the radiosensitivity.^10^ In contrast, activation of NF-κB is bound to reduce the radiosensitivity of esophageal tumor cells. *In vitro* studies have shown that suppressing the NF-κB activity can remarkably augment the radiosensitivity of several tumor cells,^11^^,^^12^ indicating that it is crucial to suppress its activity. In this study, the expression levels of TLR4 and p65 were significantly higher in the highly sensitive group than those in the lowly sensitive group, suggesting that activation of the TLR4/NF-κB signaling pathway may play an essential role in the radiation resistance and that radiotherapy in combination with NF-κB activity-inhibiting drugs may boost the sensitivity.

It is well-established that the patients with severe reactions survive longer than those insensitive, so increasing the radiosensitivity of esophageal tumor tissues is important for improving the prognosis in clinical practice. In this study, the findings showed that the activity of the TLR4/NF-κB signaling pathway was closely associated with the radiosensitivity of esophageal cancer. Presumably, this pathway may be involved in the decreased radiation resistance of tumor tissues, which still requires *in vitro* and animal model experiments.

## Authors’ Contributions:

**TH and WF:** Designed the study and revised the manuscript;

**ZXF, ZJ, CL, LJD and XEC: **Collected and analyzed the data, and prepared the manuscript.

## Notes

***S*** ***ource of funding*** ***:*** Changzhou Health Bureau Major Project of 2011 (No. ZD201114).
